# High-Throughput Sequencing Identifies MicroRNAs from Posterior Intestine of Loach (*Misgurnus anguillicaudatus*) and Their Response to Intestinal Air-Breathing Inhibition

**DOI:** 10.1371/journal.pone.0149123

**Published:** 2016-02-12

**Authors:** Songqian Huang, Xiaojuan Cao, Xianchang Tian, Weimin Wang

**Affiliations:** 1 College of Fisheries, Key Lab of Agricultural Animal Genetics, Breeding and Reproduction of Ministry of Education/Key Lab of Freshwater Animal Breeding, Ministry of Agriculture, Huazhong Agricultural University, Wuhan, 437000, Hubei, People’s Republic of China; 2 Freshwater Aquaculture Collaborative Innovation Center of Hubei Province, Hubei, People’s Republic of China; The Ohio State University, UNITED STATES

## Abstract

MicroRNAs (miRNAs) exert important roles in animal growth, immunity, and development, and regulate gene expression at the post-transcriptional level. Knowledges about the diversities of miRNAs and their roles in accessory air-breathing organs (ABOs) of fish remain unknown. In this work, we used high-throughput sequencing to identify known and novel miRNAs from the posterior intestine, an important ABO, in loach (*Misgurnus anguillicaudatus*) under normal and intestinal air-breathing inhibited conditions. A total of 204 known and 84 novel miRNAs were identified, while 47 miRNAs were differentially expressed between the two small RNA libraries (i.e. between the normal and intestinal air-breathing inhibited group). Potential miRNA target genes were predicted by combining our transcriptome data of the posterior intestine of the loach under the same conditions, and then annotated using COG, GO, KEGG, Swissprot and Nr databases. The regulatory networks of miRNAs and their target genes were analyzed. The abundances of nine known miRNAs were validated by qRT-PCR. The relative expression profiles of six known miRNAs and their eight corresponding target genes, and two novel potential miRNAs were also detected. Histological characteristics of the posterior intestines in both normal and air-breathing inhibited group were further analyzed. This study contributes to our understanding on the functions and molecular regulatory mechanisms of miRNAs in accessory air-breathing organs of fish.

## Introduction

Dojo loach (*Misgurnus anguillicaudatus*), belonging to the family Cobitidae, is widely distributed on the Eurasia continent, including Japan, South Korea and most areas of China [1]. *M*. *anguillicaudatus* can exchange gases both in water, via the gill and skin, and in air, via the posterior intestine [2]. It can swim to the water surface to breathe air though its mouth, and exchange the O_2_ and CO_2_ in the posterior intestine. Although *M*. *anguillicaudatus* is not an obligate air-breather, aerial gas exchange normally occurs even at 10°C in air-saturated water. This special characteristic suggests that *M*. *anguillicaudatus* is a potential model organism for studies of mechanisms of accessory air-breathing function in some fish.

MicroRNAs (miRNAs) are a class of small endogenous noncoding RNAs with 20–25nt, embedded within the stem regions of hairpin transcripts that exist in a wide range of invertebrates and vertebrates. miRNAs play a pivotal role in the regulation of gene expression at the post-transcriptional level, especially for signaling pathways involved in development, cellular differentiation, proliferation, apoptosis and oncogenesis [3,4]. They negatively regulate gene expression through sequence-specific interactions with the 3’untranslated regions (UTRs) of target mRNAs and thereby cause translational repression or mRNA destabilization [5,6]. Since the first discovery of the miRNA family, i.e. lin-4 and let-7, in *Caenorhabditis elegans* in 1993 [7,8], many endogenously no-encoded miRNAs have been identified in mammals, plants, insects, worms, and viruses through plasmid vector cloning, northern blotting, microarray assay and sequencing technology in recent years [9]. Currently, 28,645 mature miRNAs from 223 species have been discovered and deposited in the public available miRNA database miRBase (Newly released on 21 June, 2014). With rapid development of the next-generation sequencing technology, it is possible to precisely identify more and more novel or weakly expressed miRNAs. Some studies have identified many miRNAs from model fish species, namely zebrafish (*Danio rerio*) [10]. A few non-model fish species have also been studied for miRNAs, although mostly for growth and gonad development [11,12,13].

Because of having diverse ancestries, modern fishes that have accessory air-breathing functions show remarkable diversity in air-breathing organs (ABOs), such as skin, buccal and pharyngeal cavities, swim-bladder, digestive tract, etc [14]. Knowledges about the diversity of miRNAs and their roles in accessory ABOs of fish are still unknown. In this study, we constructed two small RNA libraries of the posterior intestines (an ABO) from normal *M*. *anguillicaudatus* (the normal group, S01) and *M*. *anguillicaudatus* with inhibited intestinal air-breathing (the inhibited group, S02). Through high-throughput sequencing and bioinformatic analysis, miRNAs in the two libraries of *M*. *anguillicaudatus* were identified and the differentially expressed miRNAs were analyzed, while potential miRNA target genes were predicted and analyzed. Histological characteristics of the posterior intestines in the normal and inhibited group were further analyzed. The discovery of miRNA resources in this study will contribute to a better understanding of miRNAs’ diversities and functions in *M*. *anguillicaudatus*, and the roles of miRNAs in regulating the biological processes of accessory air-breathing in fish.

## Results

### Histological observations of loach posterior intestines from the normal and inhibited group

**Fig 1:**
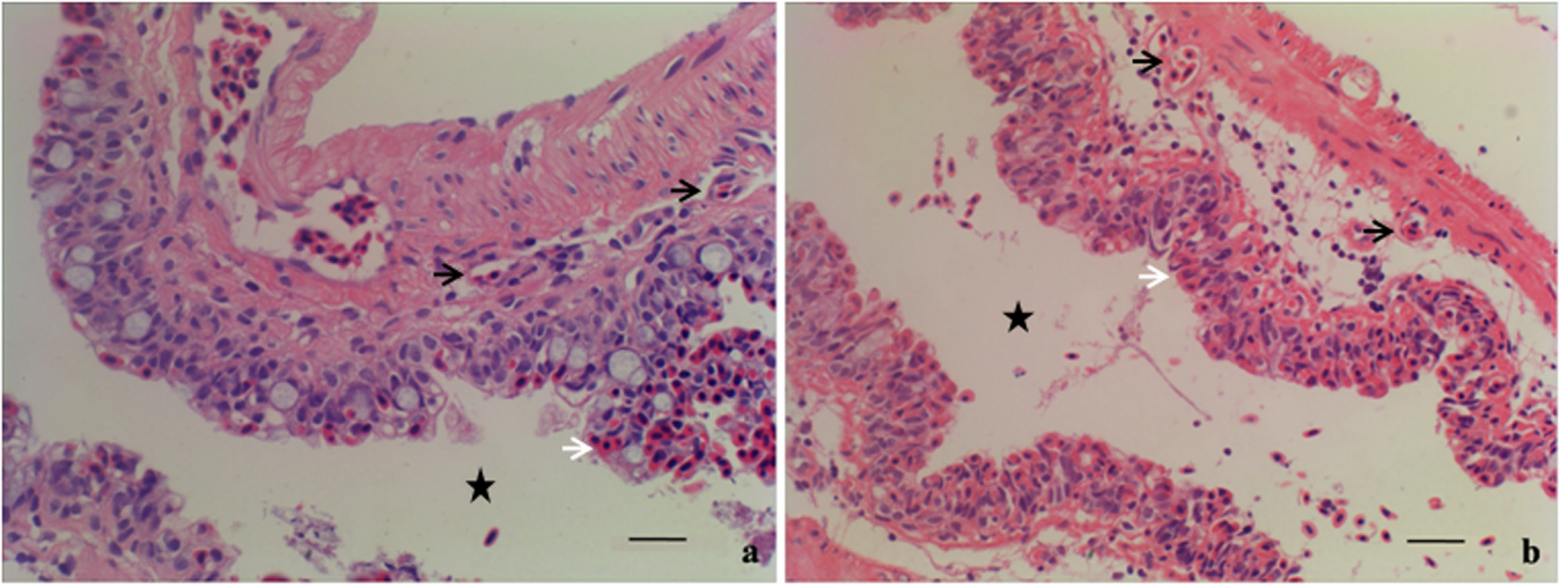
Histological structures of posterior intestines of M. anguillicaudatus. **a: the normal group (S01); b: inhibited group (S02).** Black arrows showed the intraepithelial capillaries, while white arrows showed the red blood cells (RBCs). The pentagram showed the enteric cavity. The intimal epithelium layer was broken and a big number of RBCs from the broken intimal epithelium layer were released into the enteric cavity in S02. Bar = 20μm.

**Fig 2:**
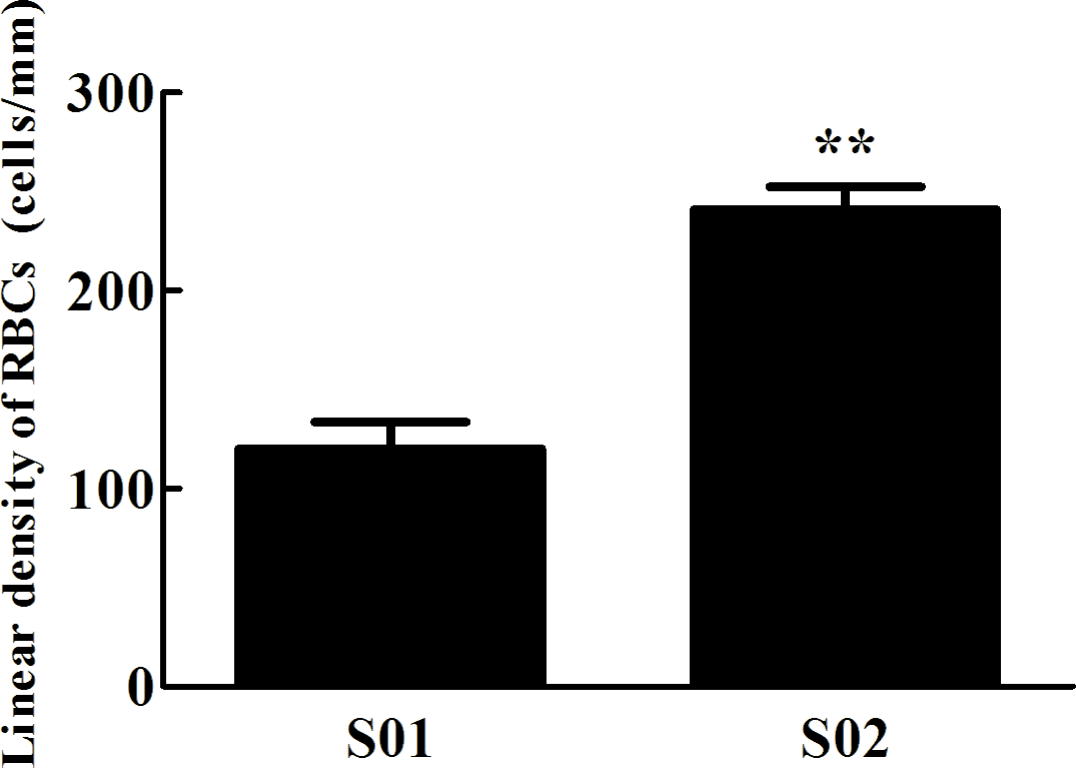
The linear density of red blood cells (RBCs) in the intimal epithelium layer of posterior intestines from the normal (S01) and inhibited group (S02). ** indicated highly significant difference between S01 and S02 group.

Loach posterior intestines from the normal group (S01) were sanguineous and gauzy, where there were lots of air bubbles. The histological observation of normal posterior intestine showed that it was well vascularized with intraepithelial capillaries and red blood cells (RBCs) nested in the intimal epithelium layer for oxygen exchange (Fig 1A). In contrast, the posterior intestines from the inhibited group (S02) showed considerable differences in morphology and histological structures. The posterior intestines from S02 were complexionless, and their histological observations showed that the intimal epithelium layer was broken and a big number of RBCs from the broken intimal epithelium layer were released into the enteric cavity (Fig 1B). The linear density of RBCs nested in the intimal epithelium layer, defined as the RBC number per millimeter, was 240 cells per millimeter from S02, significantly higher than that from S01 (Fig 2).

### High-throughput sequencing analysis of small RNAs

**Fig 3:**
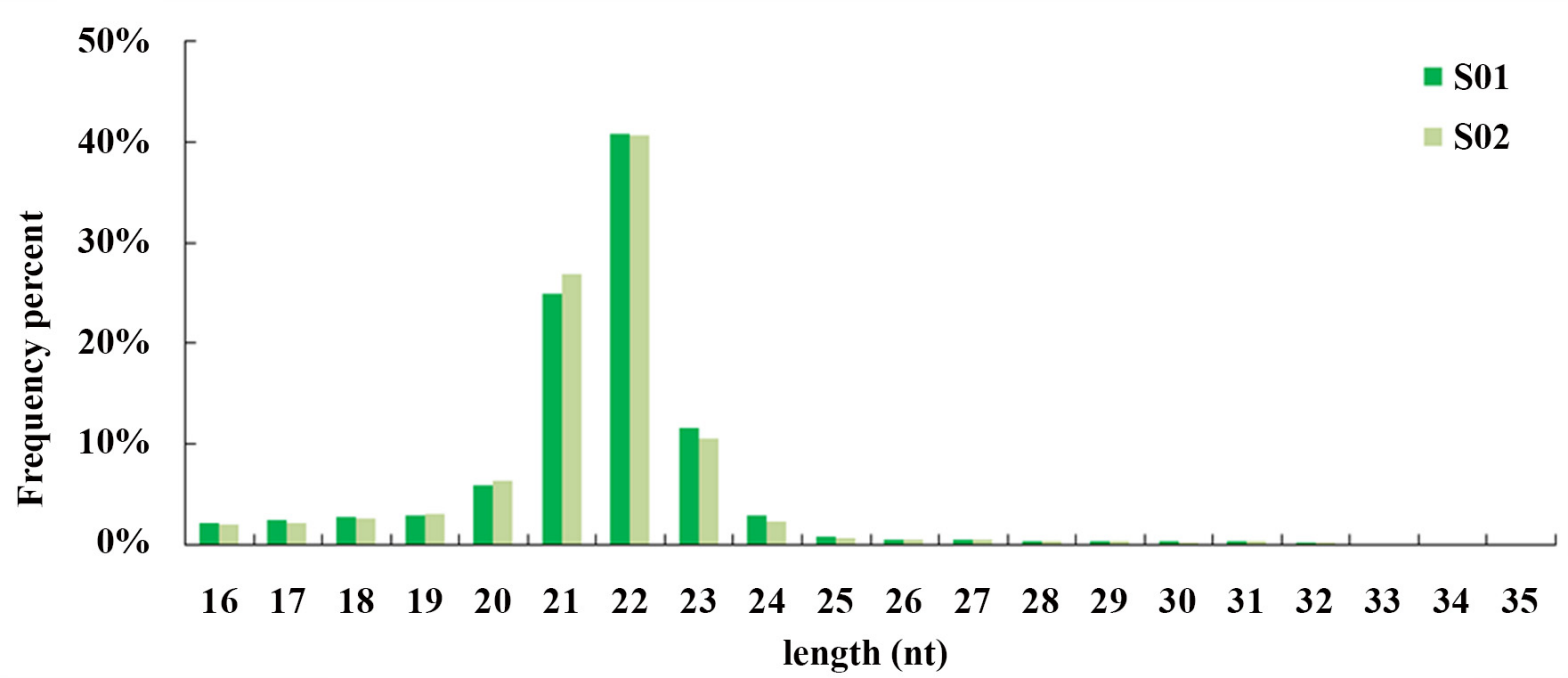
Length distributions of small RNAs in posterior intestines from the normal (S01) and inhibited (S02) group.

A total of 36,284,637 high quality reads was obtained. After removal of the adapters, reads with unknown nucleotides, reads smaller than 16 nucleotides and larger than 35 nucleotides, 30,828,574 clean reads were extracted. There were no significantly differences in length distributions of the sequences between the two libraries, with most of the sequences between 21–23 nucleotides (Fig 3).

After comparing the small RNA sequences against NCBI, RFam database and transcriptome data of *M*. *anguillicaudatus*, 2,277,016 and 2,324,552 reads of rRNA, tRNA, snRNA, snoRNA and other unannotated were removed from the S01 and S02 library, respectively. 24,950,568 reads, including 11,760,706 (83.78% of S01 clean reads) and 13,189,862 (85.02% of S02 clean reads) from the S01 and S02 library were kept, respectively.

### Identification of known and novel miRNAs in posterior intestine of *M*. *anguillicaudatus*

A read with its number larger than 5 reads was considered as a possible miRNA in *M*. *anguillicaudatus*. Among the 24,950,568 reads in total, 82,872 unique sequences in S01 library were found to be similar to known miRNAs from other species that had been deposited in the miRNABase previously. Allowing no more than two mismatches between sequences, these miRNAs represented 201 known miRNAs. Meanwhile, 87,175 unique sequences from the S02 library were screened out in the same way, and 204 known miRNAs were identified. Combining the data from the two libraries, a total of 204 unique mature miRNAs were identified, including 201 miRNAs that overlapped in the two libraries and 3 miRNAs that were detected only in the S02 library with low counts (8 reads of man-miR-71, 6 reads of man-miR-5002 and 5 reads of man-miR-5735). The read numbers of these miRNAs ranged from 1 to 4,550,356, indicating that there were not only highly expressed miRNAs, but also weakly expressed miRNAs.

Since the genome data of *M*. *anguillicaudatus* is unavailable now, the unannotated small RNAs that could be mapped to the *M*. *anguillicaudatus* transcriptome sequences were subjected to novel miRNA predicted analysis of their secondary structure, the dicer enzyme cleavage site and the minimum free energy. 84 putative novel miRNAs were predicted in the posterior intestine of *M*. *anguillicaudatus*. Of these novel miRNAs, 80 novel miRNAs were indentified in both libraries, while 1 novel miRNA (man-novel-13-3p) was identified only in the S01 library and 3 novel miRNAs (man-novel-31-3p, man-novel-57-5p and man-novel-39-3p) only in the S02 library.

**Fig 4:**
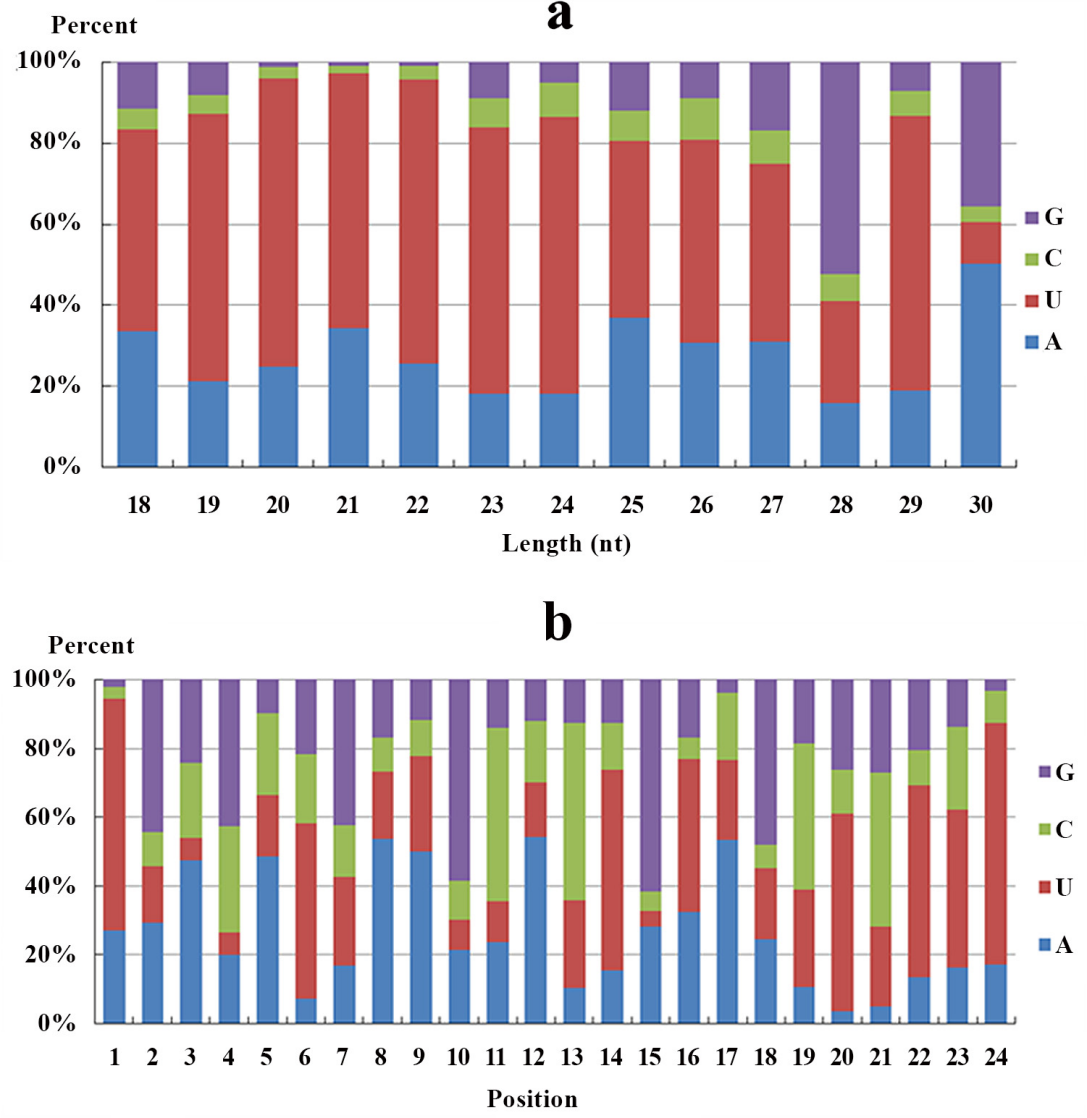
Base bias of miRNAs in *M*. *anguillicaudatus*. a: base bias on the first site of miRNAs with specific lengths; b: base bias on the specific site of miRNAs.

The base bias on the first position of identified miRNAs with a certain length and on each position of all identified miRNAs is shown in Fig 4. The majority of miRNAs tended to start with 5’-U and not with 5’-G, which was consistent with typical miRNA sequence patterns [13].

**Fig 5:**
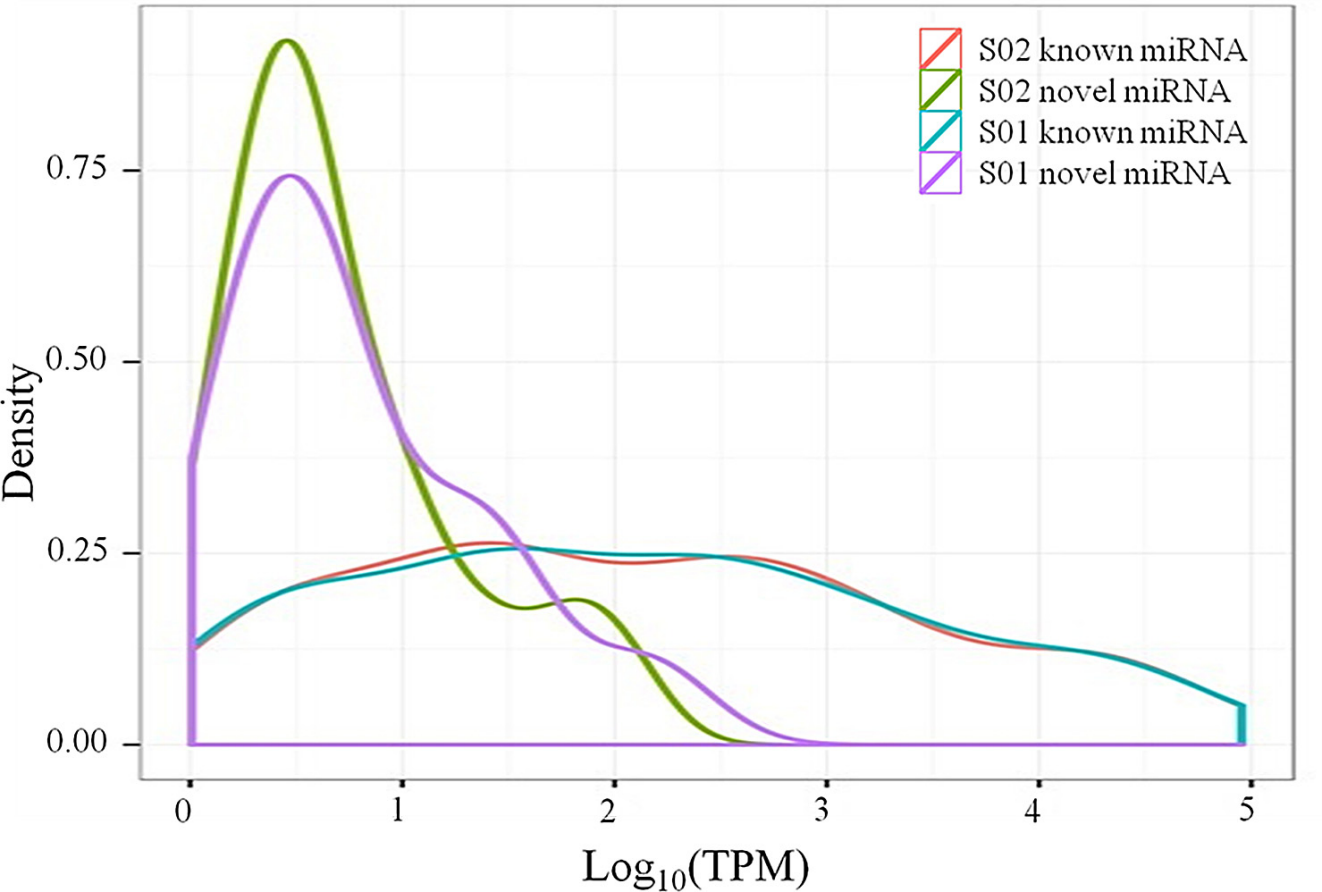
The expression quantities of known and novel miRNAs from posterior intestine in *M*. *anguillicaudatus*. S01: the normal group; S02: the inhibited group; TPM: tags per million after mapping to transcriptome.

**Fig 6:**
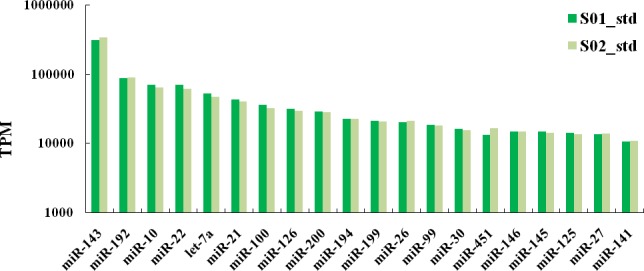
The top 20 highly-expressed miRNAs from posterior intestine in *M*. *anguillicaudatus*. S01: the normal group; S02: the inhibited group; TPM: tags per million after mapping to transcriptome.

Fig 5 shows that the novel miRNAs from the posterior intestine in *M*. *anguillicaudatus* were weakly expressed while the known miRNAs highly expressed. For the miRNAs identified here, man-miR-143 had the highest counts in both libraries, with 314,500 and 344,988 tags per million (TPM) in S01 and S02 library, respectively. The top 20 highly expressed miRNAs from the posterior intestine in *M*. *anguillicaudatus* are shown in Fig 6.

### Abundances of miRNAs in posterior intestines from the normal and inhibited group

**Fig 7:**
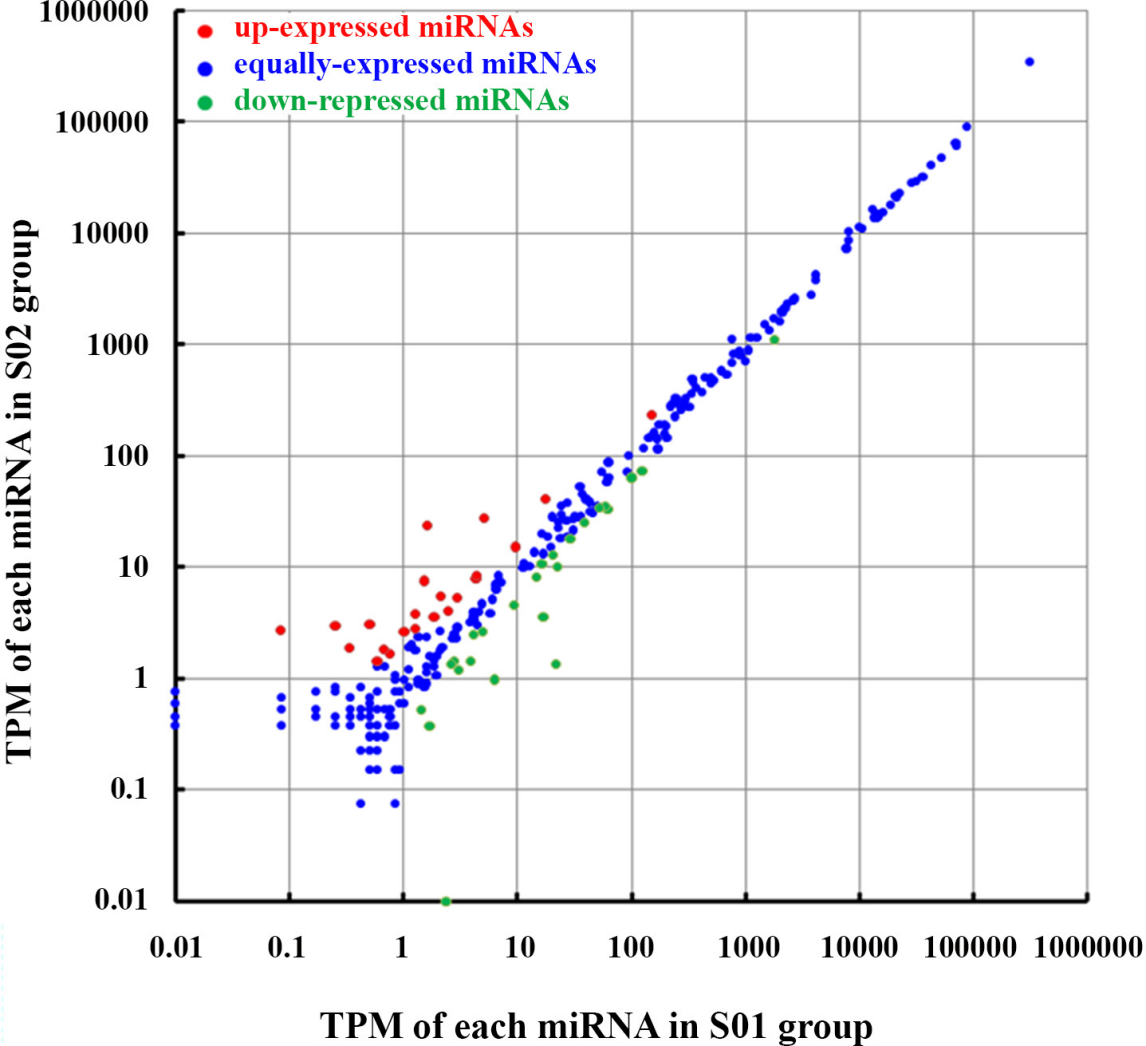
Scatter plot map for abundances of miRNAs in the normal (S01) and inhibited (S02) group. Each plot represented an individual miRNA. TPM: tags per million after mapping to transcriptome. Red dots showed up-regulated and green dots showed down-regulated miRNA in S02 (|Fold-change(log_1.5_)|>1, P <0.05).

29 known miRNAs and 18 novel miRNAs were differentially expressed between the normal and inhibited group (|Fold-change(log_1.5_)|>1, p<0.05) (S1 Table). Within the 47 differentially expressed miRNAs, 22 miRNAs and 25 miRNAs presented higher expression levels in S02 and S01 group (Fig 7), respectively.

### Prediction of potential miRNA targets genes

Within the 92,962 unigenes from our *M*. *anguillicaudatus* transcriptome data, a total of 35,091 unigenes were predicted for all the expressed miRNAs which were identified in this study. 21,804 unigenes were identified as target genes for the differentially expressed miRNAs between the two libraries. Among these target genes, 21,716, 14,347, 8,049, 5,317 and 14,435 were identified in the Nr, Swiss-Prot, KEGG, COG and GO databases, respectively. These results are shown in S2 Table. Many predicted targets were likely to be targeted by multiple miRNAs at different targeting sites. Typically, *Fibronectin* (*fn1*, c109605.graph_c0), essential for blood vessel morphogenesis, could be targeted by 12 differentially expressed miRNAs, including man-miR-206, man-miR-3963, man-miR-489, man-miR-6087, man-miR-723, man-miR-735, man-miR-7704, man-novel-1-3p, man-novel-28-3p, man-novel-32-5p, man-novel-36-5p and man-novel-42-5p.

### Function analysis of the target genes for differentially expressed miRNAs

The predicted target genes for differentially expressed miRNAs were subjected to a GO analysis that revealed 16,274, 17,910 and 18,591 genes classified into 272 cellular component ontology terms, 458 molecular function ontology terms and 1,812 biological process ontology terms. They were present in: cell 92.5%, intracellular 69.4% and organelle 56.4% of the cellular component; binding 73.6%, catalytic activity 48.1% and protein binding 25.5% of molecular functions; and cellular processes 73.7%, metabolic processes 48.1%, biological regulation 45.2% of biological processes. They also indicated that some predicted target genes were closely related with vasculogenesis.

The pathways of predicted target genes were also identified and classified according to KEGG functional annotations. Intriguingly, the most overrepresented miRNA target genes belonged to the “metabolic pathways”, which was described as a set of complex metabolic networks, such as lipid, carbohydrate, amino acid and energy metabolism. The second-most pathway was the cell cycle pathway, which was an important signaling pathway to regulate cell apoptosis and cell differentiation. Furthermore, pathways associated with purine metabolism, pyrimidine metabolism, DNA replication, glycolysis/gluconeogenesis, protein digestion and absorption, fat digestion and absorption, glycine, serine and threonine metabolism were also significantly enriched.

### miRNAs-target genes regulatory network in posterior intestine of *M*. *anguillicaudatus*

**Fig 8:**
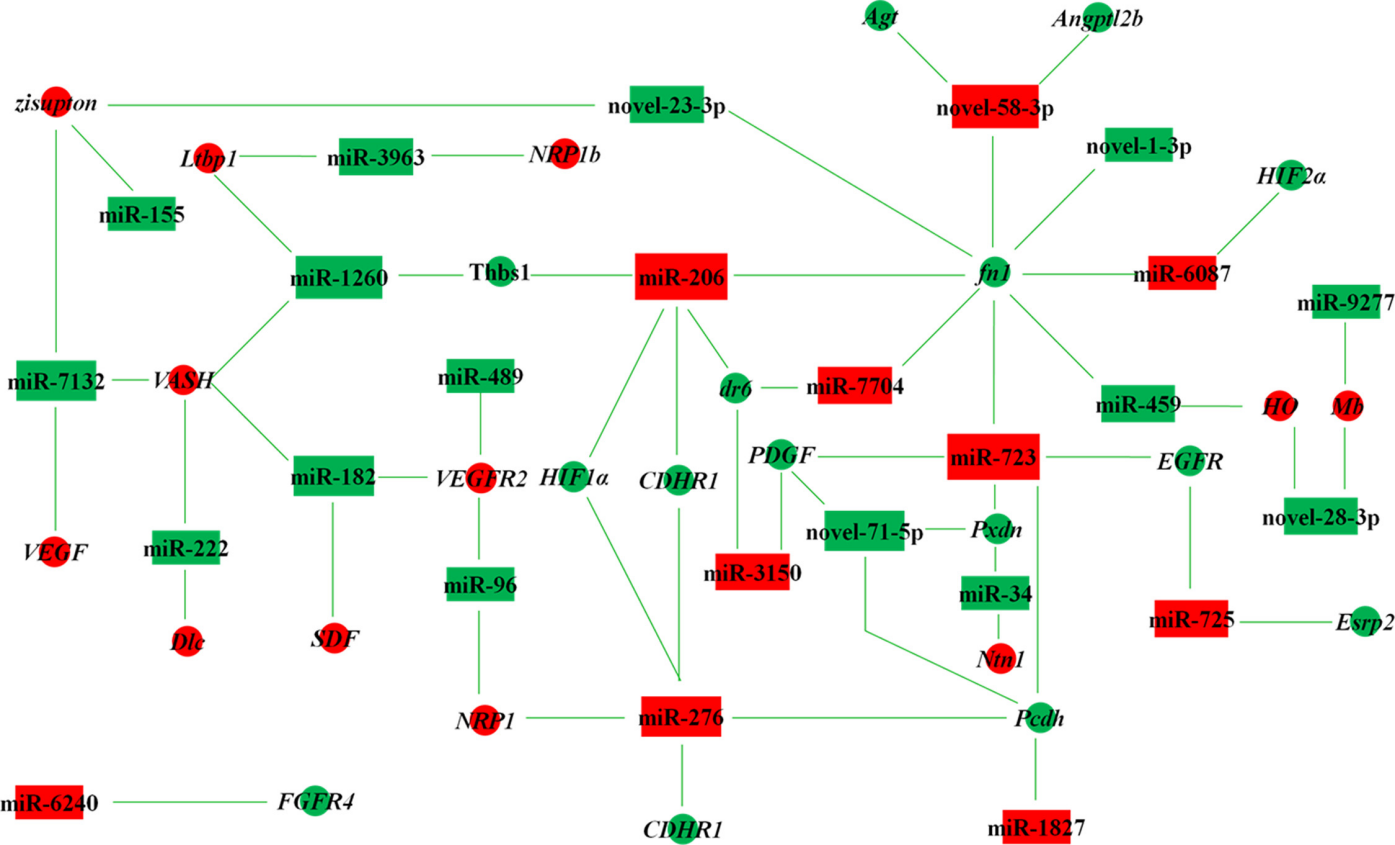
miRNAs-target genes regulatory network in posterior intestine of *M*. *anguillicaudatus*, associated with vasculogenesis and vascular structures maintenance. Boxes showed differentially expressed miRNAs between the normal and inhibited library, and circles showed the functionally related target genes of miRNAs with green lines. Red color in boxes and circles showed up-regulated and green color showed down-regulated miRNAs and genes in S02. *Agt*: Angiotensinogen; *Angptl2b*: Angiopoietin-like 2b; *CDHR1*: Cadherin 1; *Dlc*: Delta-like protein C; *dr6*: Death receptor 6; *EGFR*: Epidermal growth factor receptor; *Esrp2*: Epithelial splicing regulatory protein 2; *FGFR4*: Fibroblast growth factor receptor 4; *fn1*: Fibronectin; *HIF1α*: Hypoxia-inducible factor 1-alpha; *HIF2α*: Hypoxia-inducible factor 2-alpha; *HO*: Heme oxygenase; *Ltbp1*: Latent-transforming growth factor beta-binding protein 1; *Mb*: Myoglobin; *NRP1*: Neuropilin-1; *NRP1b*: Neuropilin-1b; *Ntn1*: Netrin-1; *PDGF*: Platelet-derived growth factor; *Pcdh*: Protocadherin; *Pxdn*: Peroxidasin; *SDF*: Stromal cell-derived factor; *Thbs1*: Thrombospondin; *VASH*: Vasohibin; *VEGF*: Vascular endothelial growth factor; *VEGFR2*: Vascular endothelial growth factor receptor 2.

29 known and 18 novel differentially expressed miRNAs and their target genes were analyzed here for establishing miRNAs-target genes regulatory network associated with vasculogenesis and vascular structures maintenance. miRNAs and their target genes with opposite counts were considered potential interactions. And then, the target genes associated with vasculogenesis and vascular structures maintenance were kept for the regulatory network’s establishment. Fig 8 shows the miRNAs-target genes regulatory network of 20 differentially expressed known miRNAs, 5 differentially expressed novel miRNAs, and 26 functionally related target genes.

### qRT-PCR analysis of miRNAs

**Fig 9:**
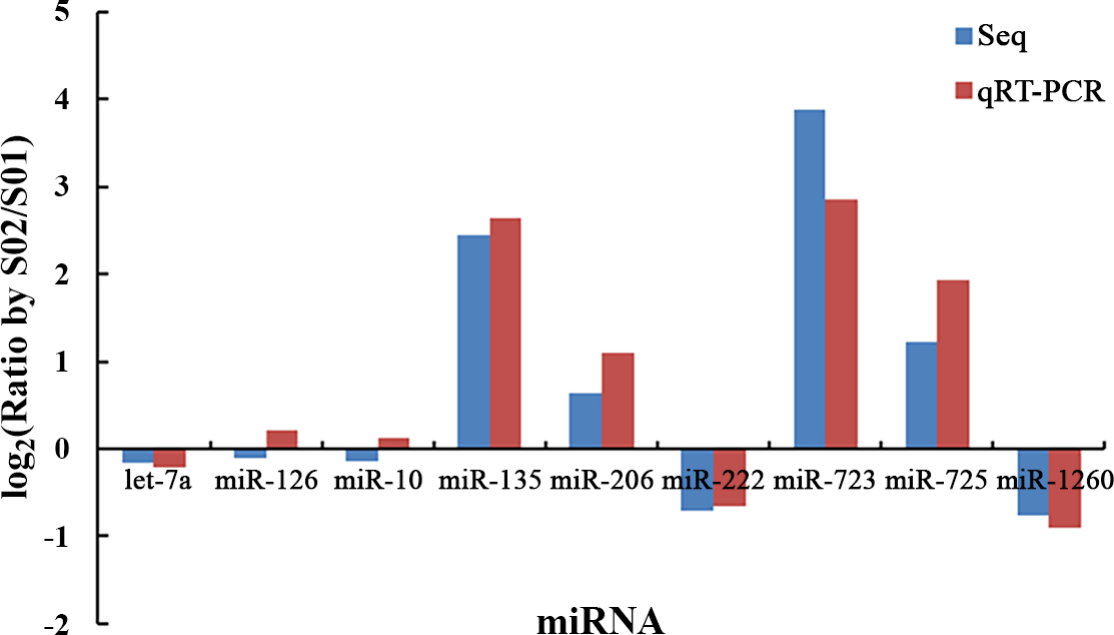
qRT-PCR validation of miRNAs expression. S01: the normal group; S02: the inhibited group; Seq: high-throughput sequencing.

Abundances of nine known miRNAs (man-let-7a, man-miR-126, man-miR-10, man-miR-135, man-miR-206, man-miR-222, man-miR-723, man-miR-725 and man-miR-1260) were validated. Except the man-miR-126 and man-miR-10, the other seven miRNAs had similar relative expressions by qRT-PCR validation when comparing with the high-throughput sequencing data (Fig 9).

### Relative expression profiles of six known miRNAs and their corresponding target genes in posterior intestines from the normal and inhibited group

**Fig 10:**
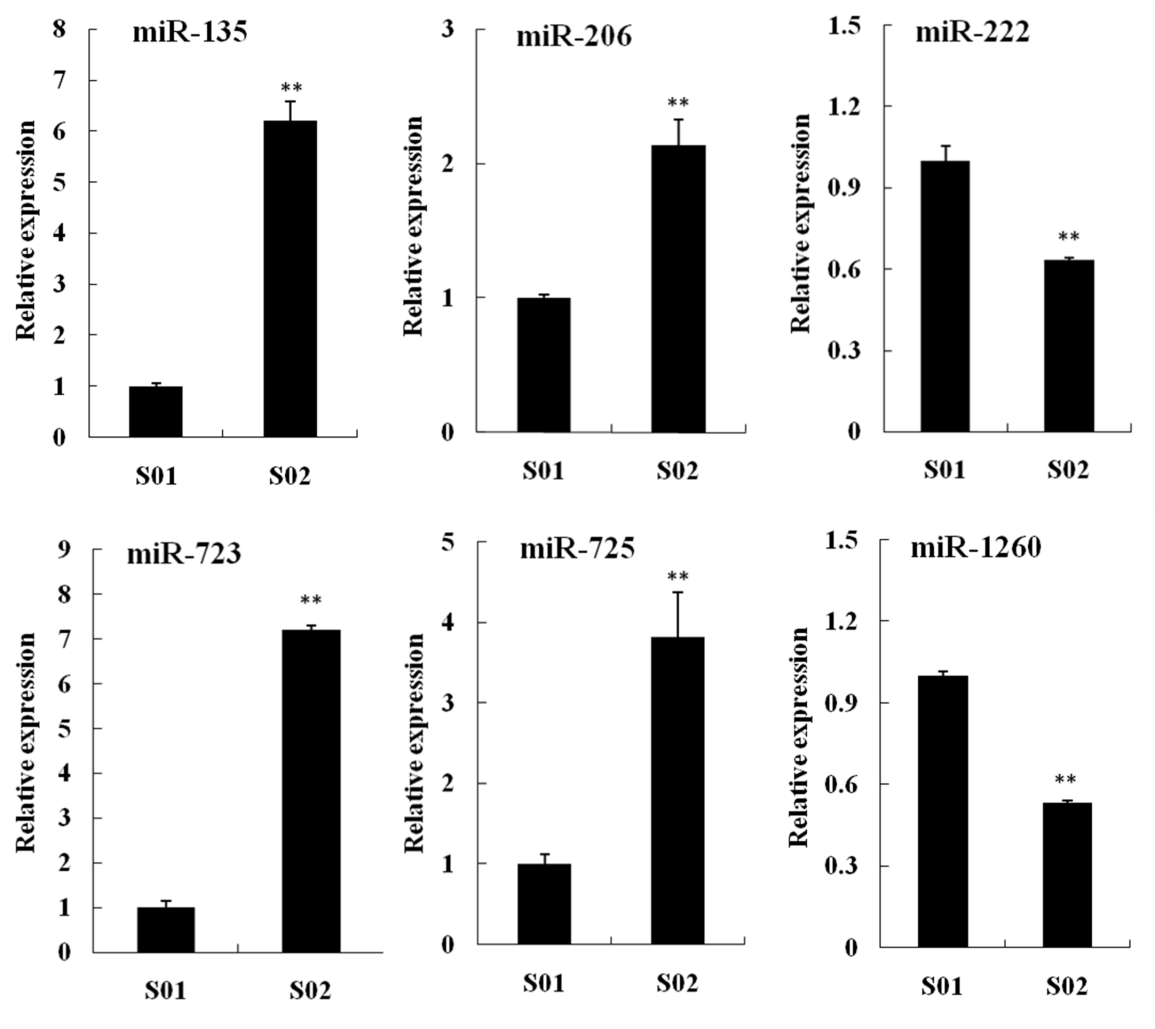
Relative expression profiles of six known miRNAs from the normal (S01) and inhibited (S02) group. Each bar represented M ± SD (n = 3). ** indicated highly significant difference between S01 and S02 group.

**Fig 11:**
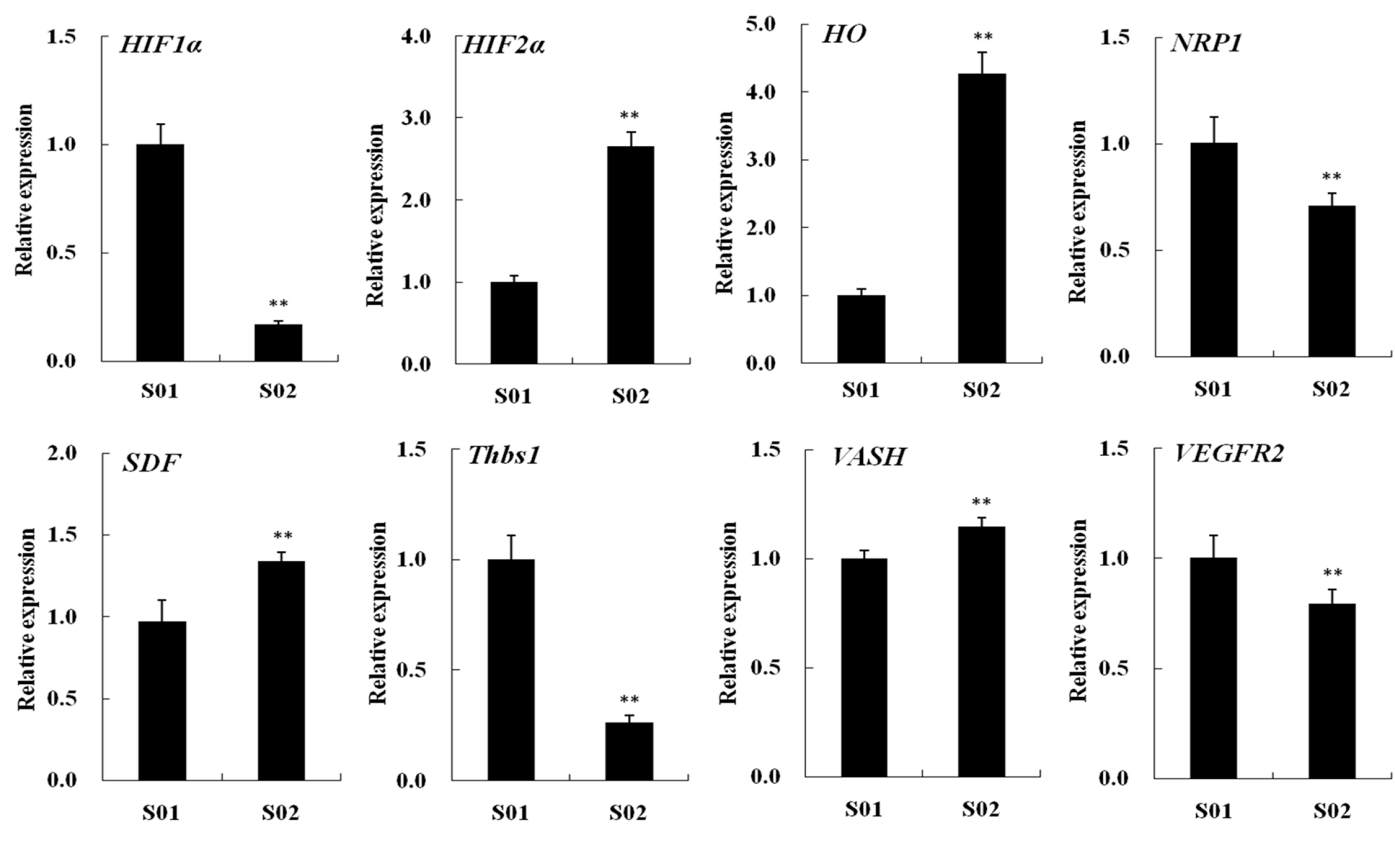
Relative expression profiles of eight corresponding target genes from the normal (S01) and inhibited (S02) group. Each bar represented M ± SD (n = 3). ** indicated highly significant difference between S01 and S02 group.

The relative expression profiles of six differentially expressed known miRNAs (man-miR-135, man-miR-206, man-miR-222, man-miR-723, man-miR-725 and man-miR-1260) and their eight corresponding target genes (*HIF1α*, *HIF2α*, *HO*, *NRP1*, *SDF*, *Thbs1*, *VASH* and *VEGFR2*) from S01 and S02 library were investigated. The relative expressions of man-miR-222 and man-miR-1260 were lower in S02 group, while other miRNAs higher (Fig 10). The relative expression levels of *HIF2α*, *HO*, *SDF* and *VASH* were significantly higher in S02 group, while the other four target genes lower (Fig 11).

**Fig 12:**
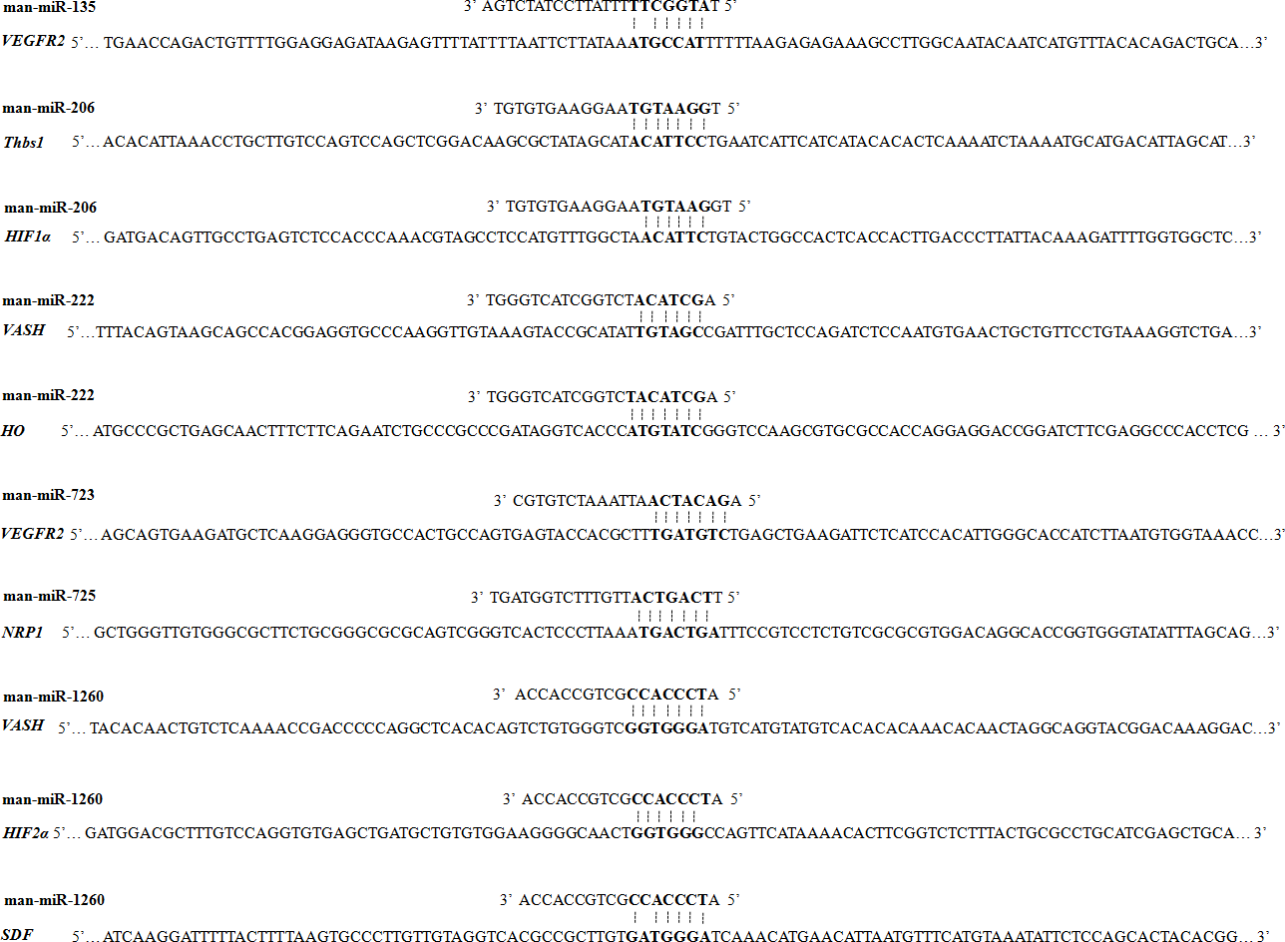
The predicted binding sites of six known miRNAs to their target genes.

There was an opposite expression between the six known miRNAs and their target genes, as man-miR-206 was higher in S02 group, while its target genes like *HIF1α* and *Thbs1* lower in the same group, and man-miR-222 and man-miR-1260 lower in S02 group, while its target genes *VASH* higher. The predicted binding sites of these miRNAs are shown in Fig 12.

### Relative expression profiles of two novel potential miRNAs in posterior intestines from the normal and inhibited group

**Fig 13:**
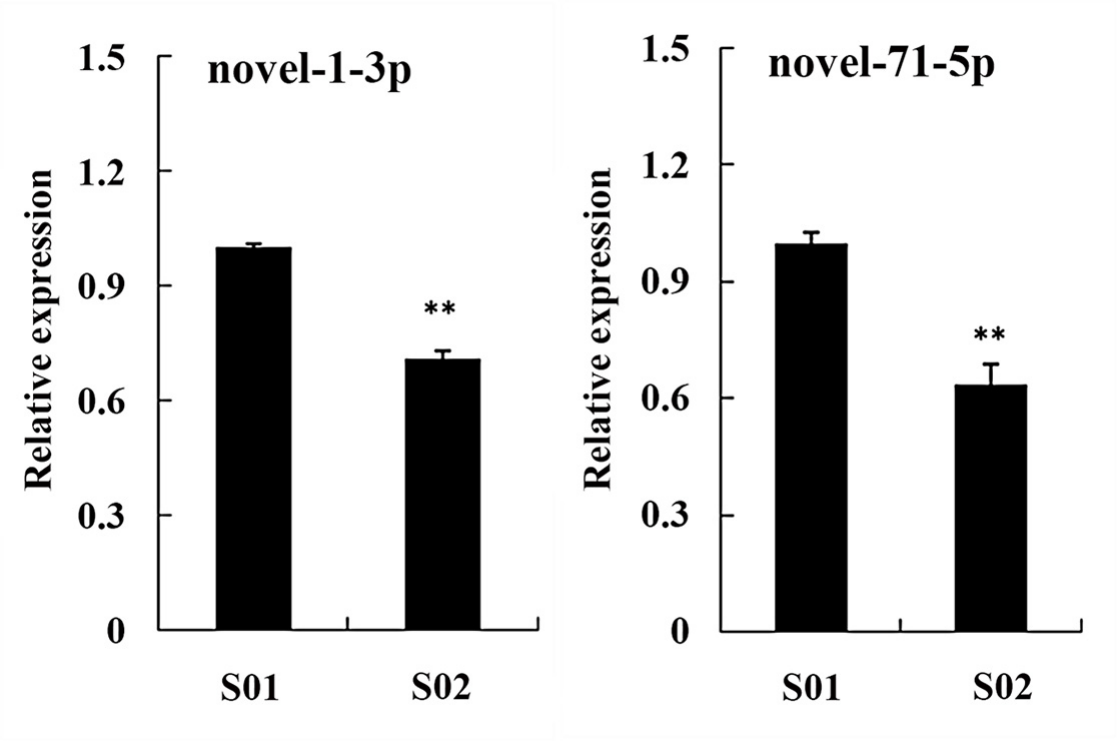
Relative expression profiles of two novel potential miRNAs from the normal (S01) and inhibited (S02) group. Each bar represented M ± SD. ** indicated highly significant difference between S01 and S02 group.

In this study, the relative expression profiles of two novel potential miRNAs with different counts (man-novel-1-3p and man-novel-71-5p) were also detected by qRT-PCR (Fig 13). The relative expression levels of the two novel potential miRNAs were significantly higher in S01 group than those in S02 group.

## Discussion

In the present study, miRNAs were identified and characterized in posterior intestines from the normal and air-breathing inhibited loach through high-throughput sequencing. It was the first time to research miRNAs in *M*. *anguillicaudatus* and also the first time to study miRNAs from an accessory air-breathing organ (ABO) of fish. A total of 204 known miRNAs and 84 novel miRNAs were detected here. Target genes prediction for miRNAs and detailed functional information were important aspects of this study. A total of 35,091 unigenes were predicted for all the expressed miRNAs identified here, and 29 known miRNAs and 18 novel miRNAs were differenttially expressed between the normal and inhibited air-breathing group. The target genes of these miRNAs were also analyzed in detailed. These results provided useful information for further research on miRNAs in *M*. *anguillicaudatus* and the functions and molecular regulatory mechanisms of miRNAs in accessory air-breathing organs of fish.

Many studies have confirmed that miRNAs play important roles in vasculogenesis, revascularization and vascular maintenance [15–18]. Table 1 summarizes most of the detected microRNAs involved in vascular biology in recent years. In the present study, 50% of the top 10 high-expressed miRNAs in posterior intestines from the normal and air-breathing inhibited group, i.e. miR-143, miR-10, miR-100, miR-126 and miR-200, was proved to be associated with vasculogenesis in previous studies, indicating the importance of miRNAs on regulating intestinal air-breathing in *M*. *anguillicaudatus*. miR-143 has been confirmed that it can regulate phenotype modulation of vascular smooth muscle cell by *TGFβ* and *BMP4* in human primary pulmonary artery smooth muscle cells (PASMCs) [19]. miR-10 regulates the angiogenic behavior and endothelial cells differentiation by promoting VEGF signaling in zebrafish [18]. miR-100 has an antiangiogenic function and represses mTOR signaling in endothelial and vascular smooth muscle cells in mice [20]. miR-126 governs vascular integrity and angiogenenic signaling though *EGFL7*, *VEGF* and *FGF* in mice [15,17,21]. Choi *et al* have reported miR-200 negatively regulates VEGF signaling in A459 cells [22].

**Table 1:** Summary of detected microRNAs involved in vascular biology.

MicroRNAs	Target genes	Functions	Reference
Pro-angiogenic microRNA			
miR-15/107 cluster	*VEGF*, *HIF-1α*,*BCL2*	mediate confounding regulation of angiogenic signaling in response to hypoxia/ischemia	[23]
miR-23~27~24 cluster	*SPRY2*, *SEMA6A*	regulators of MAPK activation	[24,25]
miR-130	*GAX*, *HOXA5*	promote angiogenesis by repressing the antiangiogenic activity of its target homeobox proteins	[26]
miR-155	*AngⅡR1*	regulate angiotensin II induced endothelial inflammation and migration	[27,28]
miR-210	*Ephrin-A3*	stimulate angiogenesis and VEGF-induced cell migration by over-expression	[29–31]
Anti-angiogenic microRNA			
miR-17/92 cluster	*Tsp1*, *CTGF*, *TGFβRⅡ*	negative regulate angiogenesis in ECs *in vitro* and *in vivo*	[32]
miR-21	*PTEN*, *Bcl-2*	decrease proliferation and increase apoptosis of VSMCs *in vitro* and in injured rat carotid artery *in vivo*	[33]
miR-100	*mTOR*	modulate proliferation, tube formation and sprouting activity of ECs and migration of VSMCs *in vitro*	[20]
miR-126	*SPRED-1*, *PI3KR2*, *VCAM-1*, *VEGF*	play important roles in angiogenesis, tumor growth and invasion, and vascular inflammation.	[15, 21,34,35]
miR-200 family	*ZEB1*, *SIP1*, *VEGF*, *VEGFR1*, *VEGFR2*, *Ets1*	play critical roles in epithelial mesenchymal transition	[22,36,37]
miR-214	*eNOS*	reduce in eNOS expression, and *in vitro* cell migration and tube formation	[38]
miR-217/34a	*SirT1*	modulate EC senescence and angiogenesis *in vitro*	[39]

In this study, the expression levels of miR-222, miR-155, miR-206, miR-489 and miR-34, etc, which involved with vasculogenesis, were significantly different expressed between the normal and air-breathing inhibited group. miR-222 presents anti-angiogenic effects [4]. Overexpression of miR-222 indirectly reduces the expression of the endothelial nitric oxide synthase (*eNOS*) in Dicer siRNA-transfected cells [40]. The miRNAs targeting *eNOS* might not only regulate angiogenesis, but might also be involved in vasculogenesis [23]. miR-155 plays a key role in the homeostasis and function of the immune system [41–43]. Over-expression of miR-155 can block endothelial cells migration in response to angiotensin α by targeting angiotensin α type 1 receptor (*AT1R*) [27,43]. miR-206 can promote muscle differentiation [44] and pulmonary artery smooth muscle cell proliferation and differentiation [45]. miR-489 has an important function of maintenance of muscle stem cell quiescence [46], and it also regulates chemoresistance in tumor cell [47,48]. Overexpression of miR-34 induces premature senescence-like phenotype and impaired angiogenesis by repressing silent information regulator 1 (*SirT1*) in endothelial cells [23]. Most of the differentially expressed miRNAs between the normal and air-breathing inhibited group have vital functions of angiogenic signaling and vascular integrity. Loach’s air-breathing function is based on enough and integrated intraepithelial capillaries distributed in its posterior intestine. The intimal epithelium layer of posterior intestine of loach from the air-breathing inhibited group was broken and the red blood cells from the broken intimal epithelium layer were released into the enteric cavity. These results indicated that these differentially expressed miRNAs involved with functions of vasculogenesis, angiogenic signaling, vascular integrity, etc, played very important roles in maintaining air-breathing function of *M*. *anguillicaudatus*.

miRNAs-target genes regulatory network associated with vasculogenesis and vascular structures maintenance was established in this study. For example, miR-206 here was up-regulated and its predicted target genes, *HIF1α*, *Thbs1* and *VEGF*, were down-regulated in the inhibited air-breathing group. These corresponding target genes were also verified with functions of regulating angiogenesis in previous studies. Bioinformatic analysis and dual-luciferase reporter gene assays revealed direct evidence for miR-206 targeting of *HIF1α* in rats’ lung tissue [49]. And miR-206 can regulate the developmental angiogenesis through modulating *VEGF* expression using target protectors in zebrafish [50].

Many novel miRNAs were also detected in the posterior intestine of *M*. *anguillicaudatus*. The significantly different expressed miRNAs, novel-1-3p, novel-23-3p, novel-28-3p, novel-58-3p and novel-71-5p, may play important roles in regulating air-breathing process in *M*. *anguillicaudatus*. But the truth is unknown until now. It is likely to be confirmed by further studies. Considering that the diversity of miRNAs and their roles in accessory air-breathing in loach remain ambiguous, this study provides useful understanding of miRNAs’ diversities and functions in *M*. *anguillicaudatus*, and miRNAs roles in regulating the biological processes of accessory air-breathing in fish.

## Methods

### Ethics

This study was conducted in strict accordance with the recommendations in the Guide for the Care and Use of Laboratory Animals of Huazhong Agricultural University. All efforts were made to minimize suffering of the animals.

### Animals and tissue collections

All experimental *M*. *anguillicaudatus* (the average weight and body length were 1.46±0.36 g and 66.93±5.70 mm, respectively) were obtained from our breeding populations for diploid *M*. *anguillicaudatus*, raised in the Aquaculture Base of College of Fisheries, Huazhong Agricultural University in Wuhan City, Hubei Province, China. All experimental procedures involving fish were approved by the institution animal care and use committee of the Huazhong Agricultural University. Sixty loaches were divided into two parts. Each part, including the normal group (S01) and intestinal air-breathing inhibited group (S02), was cultured in the same plastic tank. For making the intestinal air-breathing inhibited group (S02), we had implemented to control the range of *M*. *anguillicaudatus* activities and limited it under the water surface for 28 days. And we monitored the loaches three times a day. (The pre-experiment showed that the loaches would be died around 30 days when they were limited under water surface that they could not perform intestinal air-breathing). The posterior intestine tissues of ten surviving loaches from S02 were dissected out, while these from the normal group (S01) were also obtained for small RNA libraries constructed. The experimental loaches were cultured in a flowing water with 6.5±0.7 mg·L^-1^ dissolved oxygen and fed with tubificidae twice a day. The loaches were anaesthetized in aerated water with 100 mg·L^-1^ tricaine methanesulfonate (MS-222) and then put on ice for tissues dissections. The posterior intestine tissues were snap-frozen in liquid nitrogen for 12 h and then stored at -80°C for RNA isolation.

### Histological observations of loach posterior intestines from the normal and inhibited group

After anesthetizing, posterior intestines of loaches from S01 and S02 were dissected out and fixed at 4°C for 24 h in the Zamboni’s fixative and then stored in 70% ethanol. After dehydration in a graded series of ethanol and transparency by xylene, the posterior intestines were embedded in paraffin and sectioned in serial transverse sections (5-μm thick), using a Leica RM 2135 rotary microtome (Leica Ltd, Wetzlar, Germany). Dewaxed serial sections were stained with Delafield’s haematoxylin, counterstained with eosin (HE) to show general histological characteristics [51].

### Small RNA isolation and cDNA library constructions

Total RNA was isolated from posterior intestine tissues of the normal and inhibited group using Trizol reagent (TaKaRa, Japan) according to the manufacturer’s protocol. After an examination to assess RNAs’ qualities by NanoDrop 2000 (Thermo Scientific, Wilmington, DE, USA), RNAs from the same group were mixed together, each with equivalent concentration. Two small RNA libraries were constructed from S01 and S02 group, respectively. Small RNAs of 16–40 nt in length were firstly isolated from the total RNA by size fraction with 15% TBE urea polyacrylamide gel electrophoresis. Then, these small RNAs were ligated with 5’-RNA and 3’-RNA adapters and subsequently reverse transcription PCR was used to create cDNA. The amplified cDNA were purified and sequenced by Illumina technology.

### Small RNA sequence analysis

We first filtered low quality reads and reads containing polyA tail, reads less than 16 nt or more than 35 nt and adaptor sequences, with the aid of a dynamic programming algorithm. After filtering, statistics of the quality and length distribution of sequencing data were performed. Afterwards, a standard bioinformatics analysis of small RNA sequences was implemented. The remaining sequences were blasted against NCBI noncoding RNA database (http://blast.ncbi.nlm.nih.gov/) and Rfam database (ftp://sanger.ac.uk/pub/databases/Rfam/) to separate out rRNA, tRNA, snRNA and other ncRNA sequences, and then aligned to the transcriptome data of *M*. *anguillicaudatus* to locate on the transcriptome. Subsequently, miRNA identification was performed by comparing the sequences with the known mature miRNAs and miRNA precursor in miRBase 21.0 (http://www.mirbase.org/) to identify known miRNAs. Meantime, many unannotated sequences that cannot match any above databases were analyzed by miRDeep (http://deepbase.sysu.edu.cn/miRDeep.php) to predict novel miRNA candidates. Their hairpin structures were then analyzed using RNAfold software (http://rna.tbi.univie.ac.at/cgi-bin/RNAfold.cgi). Then we analyzed the base bias composition on the first position of identified miRNAs with certain length and on each position of all miRNAs, respectively.

### Analysis for differentially expressed miRNAs

Total miRNA reads were used to normalization to select differentially expressed miRNAs between the two libraries. The counts of miRNAs were normalized according to the tags per million (TPM). Expression levels of the known and novel miRNAs between the two libraries (the S01 group was used as the control) were compared to find out the differentially expressed miRNAs. The selection method used here was according to Audic and Claverie [52]. IDEG6 software [53] was used to analyze the different expressions of miRNAs in this study.

### Prediction of potential miRNA target genes and functional analysis

As the genome references of *M*. *anguillicaudatus* were not available, we selected the transcriptome sequences of *M*. *anguillicaudatus* obtained by our laboratory to predict miRNA target genes with the strategy described in previous studies [5,54]. Briefly, miRNAs identified in this study were used to search for antisense hits in the reference RNA sequences. Subsequently, mRNA sequences exhibiting perfect or near perfect complementarity with the corresponding miRNAs were selected and analyzed with TargetScan (http://www.targetscan.org/), mireap (https://sourceforge.net/projects/mireap/) and MiRanda (http://www.microrna.org) to predict the target sequences in *M*. *anguillicaudatus*. Then all of the target sequences were annotated using the COG, GO, KEGG, Swissprot, and Nr databases. Furthermore, the enrichment analysis of the target genes for differentially expressed miRNAs was conducted with GO database (http://www.geneontology.org/), and the gene number of each GO term was calculated. The main pathways of biochemical and signal transduction significantly associated with the predicted target genes of miRNAs were determined via a KEGG pathway analysis (http://www.kegg.jp/). The regulatory network of miRNAs and their potential target genes in the posterior intestine of *M*. *anguillicaudatus* was further analyzed.

### qRT-PCR analysis

Quantitative stem-loop RT-PCR with SYBR Green PCR Master Mix (BIO-RAD) was performed to profile the relative expressions of miRNAs in the two libraries [55], while normal qRT-PCR was used to verify the relative expressions of the target genes. Total RNA was isolated using Trizol reagent following the recommendations of the manufacturer. RNA quality and purity were assessed by using a Nanodrop 2000 spectrophotometer (Thermo Scientific, Wilmington, USA). Each RNA sample (1μg) was reverse transcribed into cDNA using the cDNA Synthesis Kit (TaKaRa, Tokyo, Japan) as manufacturer instructions. The stem-loop primer was added into small RNA reverse transcription process.

PCR assays were carried out in a quantitative thermal cycler (Bio-Rad CFX96, Hercules, CA, USA) with a 20 μl reaction volume containing 10 μl SYBR Green Real-time PCR Master Mix (Bio-Rad, Hercules, CA, USA), 2 μl of cDNA, and 0.2 μM of each primer. Primer sequences are listed in S3 Table. The thermal program included 1 min at 95°C, 40 cycles at 95°C for 10 s, 60°C for 30 s, and a melt curve step from 65°C gradually increasing 0.5°C s^-1^ to 95°C, with acquisition data every 6s. Each miRNA and gene expression quantify by qRT-PCR was presented as an n = 1 done three times, and *U6* sniRNA and *β-actin* gene were used as internal controls respectively. Ct values were represented by the mean values of three independent replicates, and the relative expression levels were calculated using the ^△△^Ct method [56]. Standard errors of mean among the replicates were also calculated.

## Supporting Information

S1 Table Abundances of differently expressed miRNAs determined by high-throughput sequencing. (DOC)

S2 Table Prediction of target genes for differently expressed miRNAs between the normal and inhibited group. (XLS)

S3 Table Primers used in this study for qRT- PCR. (DOC)
