# Supplementary material for: High-Throughput Sequencing Identifies MicroRNAs from Posterior Intestine of Loach (Misgurnus anguillicaudatus) and Their Response to Intestinal Air-Breathing Inhibition
Source: PLoS One. 2016 Feb 12;11(2):e0149123. doi: 10.1371/journal.pone.0149123 (PMC4752256; doi:10.1371/journal.pone.0149123)
Supplement: S1 Table — (DOC) [file pone.0149123.s001.doc]

**S1 Table Abundances of differently expressed miRNAs determined by high-throughput sequencing**

| **miRNA Name** | **S01-std** | **S02-std** | **Fold-change(log_1.5_ S02_std/S01_std)** | **P-value** | **Sig-lable** |
| --- | --- | --- | --- | --- | --- |
| man-miR-222 | 1822.51 | 1113.58 | -1.215 | 0.000E+00 | ** |
| man-miR-206 | 150.59 | 234.12 | 1.088 | 3.810E-51 | ** |
| man-miR-489 | 101.44 | 64.60 | -1.113 | 3.470E-24 | ** |
| man-miR-155 | 62.50 | 33.81 | -1.515 | 2.190E-25 | ** |
| man-miR-1260 | 60.12 | 35.63 | -1.290 | 6.030E-19 | ** |
| man-miR-735 | 52.80 | 34.27 | -1.066 | 1.870E-12 | ** |
| man-miR-2779 | 38.86 | 25.17 | -1.071 | 1.310E-09 | ** |
| man-miR-726 | 21.77 | 1.36 | -6.830 | 1.790E-61 | ** |
| man-miR-725 | 17.77 | 41.55 | 2.095 | 4.470E-28 | ** |
| man-miR-135 | 5.10 | 27.67 | 4.170 | 2.600E-47 | ** |
| man-miR-723 | 1.62 | 23.81 | 6.635 | 3.630E-63 | ** |
| man-miR-459 | 29.25 | 18.27 | -1.160 | 1.590E-08 | ** |
| man-miR-34 | 20.83 | 13.04 | -1.155 | 2.040E-06 | ** |
| man-miR-182 | 16.50 | 10.92 | -1.018 | 1.600E-04 | ** |
| man-miR-7132 | 14.80 | 8.26 | -1.436 | 1.320E-06 | ** |
| man-miR-124 | 9.69 | 15.31 | 1.128 | 7.480E-05 | ** |
| man-miR-96 | 4.17 | 2.50 | -1.258 | 2.245E-02 | * |
| man-miR-9226 | 3.91 | 1.44 | -2.464 | 1.281E-04 | ** |
| man-miR-9277 | 3.06 | 1.21 | -2.283 | 1.413E-03 | ** |
| man-miR-6937 | 2.98 | 5.31 | 1.427 | 4.459E-03 | ** |
| man-miR-3963 | 2.81 | 1.44 | -1.644 | 1.884E-02 | * |
| man-miR-7548 | 2.13 | 5.46 | 2.326 | 1.810E-05 | ** |
| man-miR-6240 | 1.87 | 3.56 | 1.589 | 1.101E-02 | * |
| man-miR-276 | 1.53 | 7.58 | 3.946 | 3.310E-13 | ** |
| man-miR-3150 | 1.28 | 3.79 | 2.687 | 7.470E-05 | ** |
| man-miR-7547 | 1.28 | 2.81 | 1.944 | 8.027E-03 | ** |
| man-miR-6087 | 1.02 | 2.65 | 2.357 | 2.766E-03 | ** |
| man-miR-7704 | 0.77 | 1.67 | 1.921 | 4.511E-02 | * |
| man-miR-1827 | 0.68 | 1.82 | 2.427 | 1.197E-02 | * |
| man-novel-52-5p | 0.09 | 2.73 | 8.556 | 2.110E-09 | ** |
| man-novel-50-3p | 0.26 | 2.96 | 6.043 | 2.370E-08 | ** |
| man-novel-4-3p | 0.51 | 3.11 | 4.457 | 7.480E-07 | ** |
| man-novel-53-5p | 0.34 | 1.90 | 4.237 | 2.038E-04 | ** |
| man-novel-58-3p | 0.60 | 1.44 | 2.180 | 4.060E-02 | * |
| man-novel-41-3p | 4.42 | 8.34 | 1.565 | 1.102E-04 | ** |
| man-novel-32-5p | 4.34 | 7.96 | 1.498 | 2.718E-04 | ** |
| man-novel-36-5p | 2.47 | 4.09 | 1.250 | 2.619E-02 | * |
| man-novel-1-3p | 124.99 | 74.75 | -1.268 | 1.420E-36 | ** |
| man-novel-20-3p | 5.02 | 2.65 | -1.571 | 2.434E-03 | ** |
| man-novel-61-5p | 2.64 | 1.36 | -1.624 | 2.436E-02 | * |
| man-novel-23-3p | 9.52 | 4.62 | -1.781 | 3.390E-06 | ** |
| man-novel-71-5p | 22.45 | 10.24 | -1.937 | 2.080E-14 | ** |
| man-novel-42-5p | 1.45 | 0.53 | -2.471 | 2.105E-02 | * |
| man-novel-28-3p | 1.70 | 0.38 | -3.702 | 9.290E-04 | ** |
| man-novel-5-3p | 17.01 | 3.64 | -3.803 | 1.890E-27 | ** |
| man-novel-81-3p | 6.38 | 0.99 | -4.605 | 1.240E-13 | ** |
| man-novel-13-3p | 2.38 | 0.01 | -13.497 | 6.730E-10 | ** |

**Note:**

(1) S01-std: Normalized expression level of miRNA in the small RNA library of normal group;

(2) S02-std: Normalized expression level of miRNA in the small RNA library of air-breathing inhibited group;

(3) Fold-change(log_1.5_ S02-std/S01-std): Fold change of miRNAs in pair of samples;

(4) P-value: p value which reflected the significance of miRNA differential between samples;

(5) Sig-lable: significance label.

*: fold-change(log_1.5_ S02-std/S01-std)＞1 or fold-change(log_1.5_ S02-std/S01-std)＜-1, and 0.01≤ p-value＜0.05

**: fold-change(log_1.5_ S02-std/S01-std)＞1 or fold-change(log_1.5_ S02-std/S01-std)＜-1, and p-value＜0.01
