# Supplementary material for: High-Throughput Sequencing Identifies MicroRNAs from Posterior Intestine of Loach (Misgurnus anguillicaudatus) and Their Response to Intestinal Air-Breathing Inhibition
Source: PLoS One. 2016 Feb 12;11(2):e0149123. doi: 10.1371/journal.pone.0149123 (PMC4752256; doi:10.1371/journal.pone.0149123)
Supplement: S3 Table — (DOC) [file pone.0149123.s003.doc]

**S3 Table Primers used in this study for qRT- PCR**

**S3**-1 Table Primers used in this study for miRNA qRT- PCR

| **miRNA name** | **Stem loop primer 5'-3'** | **Forward sequence** |
| --- | --- | --- |
| man-let-7 | CTCAACTGGTGTCGTGGAGTCGGCAATTCAGTTGAGAACTATAC | ACACTCCAGCTGGGTGAGGTAGTAGGTTGT |
| man-miR-126 | CTCAACTGGTGTCGTGGAGTCGGCAATTCAGTTGAGTGCATTAT | ACACTCCAGCTGGGTCGTACCGTGAGTAAT |
| man-miR-10 | CTCAACTGGTGTCGTGGAGTCGGCAATTCAGTTGAGACAAATTC | ACACTCCAGCTGGGTACCCTGTAGAACCGA |
| man-miR-222 | CTCAACTGGTGTCGTGGAGTCGGCAATTCAGTTGAGACCCAGTA | ACACTCCAGCTGGGAGCTACATCTGGCTA |
| man-miR-206 | CTCAACTGGTGTCGTGGAGTCGGCAATTCAGTTGAGACACACTT | ACACTCCAGCTGGGTGGAATGTAAGGAA |
| man-miR-1260 | CTCAACTGGTGTCGTGGAGTCGGCAATTCAGTTGAGTGGTGGCA | ACACTCCAGCTGGGATCCCACCGCTG |
| man-miR-725 | CTCAACTGGTGTCGTGGAGTCGGCAATTCAGTTGAGACTACCAG | ACACTCCAGCTGGGTTCAGTCATTGTTTCT |
| man-miR-135 | CTCAACTGGTGTCGTGGAGTCGGCAATTCAGTTGAGTCAGATAG | ACACTCCAGCTGGGTATGGCTTTTTATTCCT |
| man-miR-723 | CTCAACTGGTGTCGTGGAGTCGGCAATTCAGTTGAGGCACAGAT | ACACTCCAGCTGGGAGACATCAATTAAAT |
| man-novel-1-3p | CTCAACTGGTGTCGTGGAGTCGGCAATTCAGTTGAGATCAAAGT | ACACTCCAGCTGGGTTCGCTCTCGCAGTAC |
| man-novel-71-5p | CTCAACTGGTGTCGTGGAGTCGGCAATTCAGTTGAGTCTCGAAT | ACACTCCAGCTGGGTACCGCGAGAACGAT |
| Reverse sequence | TGGTGTCGTGGAGTCG | |

**S3**-2 Table Primers used in this study for potential target genes qRT-PCR

| **gene name** | **Forward sequence** | **Reverse sequence** |
| --- | --- | --- |
| *HIF1a* | AGTTCTGCTATGCTTTGGAC | TTGCGACTGTTGTAGATGAC |
| *HIF2a* | TGACTTGAGGTTGACGGTGC | CTCTGGGCGGCTTCATTAC |
| *HO* | CTGGAGTATTTCTACGGTC | CTAACAGGTATTCAGGGTG |
| *NRP1* | TCTACTCACTTGACCCCAT | AGTTGTAGTTCTCCAGCGT |
| *SDF* | GTTCTGGCGATGGTGGCTCTT | ATTTGGTCTTGGGATTGATGC |
| *Thbs1* | TGAGAAATCACCCTGTCCAA | CACACTCTTTACCGCCATACT |
| *VASH* | TCCATGTCAGTGCCTGATTG | CAGACCGTTGGTTAGGTAGATTC |
| *VEGFR2* | TATGCCAATGGACGGACACT | ACTTGACCAAATGCTCCTCTGC |
